# Investigating pharmacy students’ therapeutic decision-making with respect to antimicrobial stewardship cases

**DOI:** 10.1186/s12909-022-03542-0

**Published:** 2022-06-17

**Authors:** Ziad G. Nasr, Diala Al Haj Moustafa, Sara Dahmani, Kyle J. Wilby

**Affiliations:** 1 College of Pharmacy, QU Health, Qatar University, PO Box 2713, Doha, Qatar; 2 College of Pharmacy, Dalhousie University, Halifax, Canada

**Keywords:** Antimicrobial Stewardship, Pharmacy education, Clinical reasoning, Think-aloud, Qualitative research

## Abstract

**Background:**

Antimicrobial stewardship programs (ASPs) play a big role in minimizing antimicrobial resistance. Pharmacists are essential members of the health care team and in order for them to fulfill roles on ASP teams and become antimicrobial stewards, they must be prepared adequately by pharmacy schools prior to entry into actual practice. Although programming has been implemented into entry-to-practice programs worldwide, little is known about how students interpret antimicrobial stewardship (AMS) data and arrive at clinical decisions. We aimed to explore students’ cognitive processes and determine how they formulate therapeutic decisions when presented with AMS cases.

**Methods:**

This was a qualitative study conducted using a case study approach, in which a sample (n=20) of pharmacy students was recruited to interpret AMS cases. Semi-structured 1-on-1 interviews were arranged with each participant. A think-aloud procedure with verbal protocol analysis was adopted to determine students’ decision-making processes. Thematic analysis was used to interpret themes from the interview data.

**Results:**

Two themes were interpreted from the data: students’ focus and students’ approach to case interpretation. Students’ focus relates to external factors students consider when interpreting AMS case data and use to make and justify therapeutic decisions including patient-centered factors, drug-related factors, AMS interventions, and pharmacist’s role. Students’ clinical reasoning describes the approach that students use to interpret the data and the decision-making processes they employ to arrive at a clinical decision including a systematic approach versus non-systematic approach.

**Conclusions:**

Students vary in their focus and the cognitive strategies used to interpret AMS cases. Findings support the notion that clinical reasoning and decision-making should be explicitly taught in pharmacy curricula, in order to help students become aware of their own cognitive processes and decision-making abilities.

## Introduction

Antimicrobial resistance (AMR) continues to be a growing public health threat [1]. The Centers for Disease Control and Prevention (CDC) reports that more than 2.8 million antibiotic-resistant infections occur yearly in the United States and more than 35,000 people die consequently, the latter which necessitates a rapid action for containment [2]. One of the strategies adopted by health care institutions to minimize AMR is the implementation of antimicrobial stewardship programs (ASPs) [3]. In order for pharmacists to adequately fulfill necessary roles on ASP teams and become antimicrobial stewards, pharmacy schools must adequately prepare student pharmacists during undergraduate and postgraduate studies prior to entry into actual practice [3, 4]. The World Health Organization stresses that future healthcare professionals should receive appropriate education on proper antimicrobial use, infection control and containment of AMR [5]. Although programming has been implemented into entry-to-practice programs worldwide, little is known about how students interpret AMS data and arrive at clinical decisions.

Clinical reasoning is a key component in providing patient-centered care, a skill needed for all healthcare professionals including pharmacists [6, 7]. The clinical reasoning process requires students to state their goals, identify a clinical question, identify their assumptions and point of view, consider relevant patient data, look into relevant guidelines and current literature, and consider factors affect their selection of drug therapy [8]. Several models were developed to describe a systematic approach towards clinical decision making [9–11]. Many educational techniques have been implemented within curricula to teach students such skills, including problem-based learning, clinical presentations, among others [12]. Several studies emphasized that clinical reasoning can help regulate antimicrobial prescribing decisions and enhance knowledge of appropriate antibiotic use [13–18]. Despite the clear need to teach students clinical reasoning, no gold standard for developing these skills has emerged [12]. It is of great interest to know what information is concentrated on by students when faced with an AMS case, and how is that information used to facilitate case problem solving. Understanding students’ processing in the interpretation of such data will aid in the design of new approaches to implement AMS theory into the curriculum. Also, it helps design robust methods that incorporate students’ skills to support judgement and decision making during AMS training in clinical practice. We therefore aimed to explore students’ cognitive processes and determine how they formulate therapeutic decisions when presented with AMS cases.

## Methods

### Study setting

The study was conducted at the College of Pharmacy at Qatar University in Doha, Qatar. The college maintains a Bachelor of Science in Pharmacy (1 pre-year and 4 professional years) and a Doctor of Pharmacy (PharmD) (1 optional post-graduate year) programs both accredited by the Canadian Council for Accreditation of Pharmacy Programs (CCAPP). The Infectious Diseases (ID) module is usually completed during the spring semester of the 3^rd^ professional undergraduate year.

### Study design

This was a qualitative study conducted using a case study approach, in which a sample of students was recruited to interpret AMS cases. A think-aloud procedure with verbal protocol analysis was adopted to capture how students brought meaning to case interpretation and solving [19].

### Study participants

All students from 4^th^ professional year and PharmD program were sent an email invite to participate in the study. After receiving confirmation, 10 students from the 4th^th^ professional year and 10 students from the PharmD program were randomly selected to participate in the study. Participants were chosen using purposive sampling based on the following characteristics: students who have finished their ID module or prior experiential training experience with patients requiring AMS decisions.

### Data collection and analysis

The primary investigator (PI), who is a clinical assistant professor at the college of pharmacy and a board certified pharmacotherapy specialist and ID pharmacist, developed three AMS cases that include stewardship strategies to be utilized by students in order to interpret the case. The cases were peer-reviewed by another board certified infectious diseases pharmacist and antimicrobial stewardship specialist from the United States. Cases and questions can be found in Table 1. Two collaborating investigators sent an AMS refresher to the participants 1 week prior to the interviewing process so that they get familiar with the content they are expecting while interpreting the cases. The refresher included few slides explaining the principles and elements of AMS, and recommendations for successful implementation of ASPs based on guidelines by the Infectious Diseases Society of America and the Society for Healthcare Epidemiology of America [20]. This step was implemented to ensure participants’ thought processes were not disrupted by being unsure of the case material encountered.

**Table 1:** Case vignettes and questions asked

Case vignettes	Questions
Case 1: A 60-year-old man (height 72 inches, weight 100 kg) with non-Hodgkin lymphoma requiring chemotherapy presents with fever (temperature in the emergency department 39.6°C), rigors, chills, and overall poor appetite. On examination of his catheter, notable erythema is around the catheter site. Paired blood cultures are obtained both centrally and peripherally. Twelve hours after admission, all four cultures grow gram-positive cocci in clusters. One month previously, he was treated for a methicillin-resistant Staphylococcus aureus (MRSA) skin/soft tissue abscess with trimethoprim/sulfamethoxazole and incision/drainage. After removing the catheter, the team starts him on Vancomycin 15–20 mg/kg intravenously every 8 hours. His most recent serum creatinine is 0.9 mg/dL.	What pharmacokinetic/pharmacodynamics principles would you implement to optimize the patient’s therapeutic regimen?
Case 2: A 58-year-old woman with a history of recurrent urinary tract infections, presents with acute pyelonephritis. She has an allergy to penicillin (unknown), and sulfa drugs. She has no other comorbid conditions. She is placed on meropenem 1 g intravenously every 8 hours for initial treatment in the hospital. Her urine culture on hospital day 3 shows more than 100,000 *Escherichia coli* with the following susceptibilities (S=Sensitive, I=Intermediate, R=Resistant): *Ampicillin/sulbactam* ***R****, cefepime* ***S****, ceftriaxone* ***S****, ciprofloxacin* ***S****, ertapenem* ***R****, gentamicin* ***I****, meropenem* ***R****, piperacillin/tazobactam* ***S****, and trimethoprim/sulfamethoxazole* ***S****.*	What management strategies would be best from two stewardship standpoints? When would you consider stepping down to oral therapy appropriate? Justify your answer while providing a drug of choice.
Case 3: This is a 186-bed hospital that has had a stewardship program for 2 years. Carbapenems use has decreased from prior efforts, yet rates of *Clostridioides difficile* infection are increasing. There is widespread use of other antipseudomonal beta-lactams empirically at this institution for unnecessary reasons, particularly if patients are doing well on them; once the antibiotic is started, it is hard to get it stopped.	What stewardship intervention is highly recommended for guiding appropriate empiric therapy selection for a patient with a specific infectious disease? Justify your answer.

Due to the ongoing COVID-19 pandemic, interviews were scheduled and conducted over ZOOM™ online platform. Each of the 2 co-investigators scheduled a meeting with one student at a time (10 students each) with the purpose of completing a think aloud protocol. Participating students were briefed about the study objectives and the think-aloud procedure. Each participating student was given three cases to interpret over a total of 20 minutes. After reading the case, the student was instructed to start interpreting the case and to verbalize all thoughts and answers loudly as they emerge. The student was prompted at all times to “keep thinking aloud” if they stopped verbalizing thoughts for more than ten seconds. The same process was repeated for all the other 19 participants. All think-aloud protocols were transcribed verbatim by two collaborating investigators (DA, SD) and checked for errors by the senior PI (ZN). The senior researcher trained the other two investigators to code transcripts using qualitative research textbooks and examples from primary literature. Transcripts were read multiple times before coding. The independent coders coded the transcripts inductively using Microsoft Word®. Codes were assigned using an open coding procedure to identify segments that relate to the research questions, and that reflect students’ approaches to interpreting, analyzing, and solving the AMS cases. Once all coding was complete, the three investigators met regularly to reconcile discrepancies and produce the final coding framework. This was sent to a fourth investigator (KJ) who is expert in qualitative research for review. Afterwards, all investigators began to categorize related codes based on similar meanings. Then, the research team met to interpret themes from the categorized data. This process occurred over a 2 months period. All investigators agreed upon final themes and supporting data.

The research team regularly met for consensus. We accounted for reflexivity in our qualitative research where it was important to recognize the perspective of all authors who interpreted the results. Two authors (DM, SD) are undergraduate pharmacy students who undertook extensive research-related courses, done clinical training, and got taught infectious diseases principles. One author is an expert in educational research and qualitative research (KW). The senior investigator (ZN) is an expert in infectious diseases clinical practice. All these background experiences may have influenced how results were analyzed. In any instances of disagreements among the research team, an open discussion took place regarding interpreting results before coding an idea or developing a theme. The thematic analysis followed a list of codes grouped upon by consensus.

## Results

We found that students focused on different aspects of AMS during the case review and that they used differing thought processes and strategies as they attempted to make sense of the data provided. Two themes were interpreted from the data: students’ focus and students’ clinical reasoning.

### Theme 1: Students’ focus

This theme focuses on the main external factors that students consider when interpreting AMS case data to prevent AMR and promote appropriate use of antibiotics while making and justifying therapeutic decisions. These factors are labeled as external to the students, as they do not represent a specific process or strategy that students use to interpret data. Instead, they are the aspects that come into the students mind when seeking to arrive at and justify their decisions. Four categories (Table 2.) were identified as part of this theme. These included:

- **Patient-centered factors:** Those are factors taken into account while interpreting AMS cases including patient’s allergy status (e.g. recommending alternative therapy in case of penicillin allergy); (quote 2.1), concomitant comorbidities, organ dysfunction (e.g. for dose adjustment), infection history, and hemodynamic stability (e.g. switching to oral therapy); (quote 2.2), and patient safety (quote 2.3).
- **Drug-bug related factors:** Those are factors that guide decision making regarding treatment recommendation including: relying on hospital’s local antibiogram (quote 2.4), interpreting culture and susceptibility report (e.g. de-escalation to narrow spectrum antibiotic); (quote 2.5), and drug safety (quote 2.5).
- **AMS interventions:** Those are important in order to decrease AMR and enhance the appropriate use of antibiotics. Examples include formulary restriction and preauthorization, prospective audit with feedback, and streamlining of drug therapy (quotes 2.6 and 2.7).
- **Pharmacist’s role:** This is important to monitor for the safety and efficacy of drug therapy (e.g. following up on patient’s signs and symptoms, ensuring optimal duration of therapy, tracking adverse effects, performing therapeutic drug monitoring); (quote 2.8), and to provide education to the multidisciplinary team (quote 2.9).

**Table 2:** Sub-categories within students’ focus

Categories	Reflective quotes
*Category 1: Patient-centered factors*	*2.1. “One strategy that I would be thinking for this patient is that, since she has an allergy to penicillin and it says it is unknown so we need to go and talk to the patient, take a history of allergy. It is very important to know about the time, because if the allergy was more than 10 years ago, then if we would ask, assess and re-challenge, we will most likely, more than 95% of patients will not have allergies to penicillin”* – Participant 5 *2.2. “I will convert from intravenous to oral once the patient is stable. Pyelonephritis is kind of a severe case, so I will have to check that the patient is totally stable and all the vitals are totally stable. And if I can minimize the hospital stay, it will be much better to convert from IV to oral to decrease any probability of getting an infection during the hospital stay”* – Participant 13 *2.3. “As for pharmacokinetic/pharmacodynamics principles, we will need to calculate the dose based on his weight and assess his kidney function in order to make any dose adjustments for his renal clearance”* – Participant 13
*Category 2: Drug-bug related factors*	*2.4. “We need to tailor our empiric therapy to the institutional resistant patterns and national antibiogram”* – Participant 2 *2.5. “We have the cultures, the sensitivities, we have the bacteria, meropenem is too broad and it covers Pseudomonas, and our bacteria appears to be a gram negative bacteria which is E.coli so we need to narrow down our choices”* – Participant 4
*Category 3: AMS interventions*	*2.6. “What I would do is that, if there is no preauthorization for a specific antipseudomonal beta-lactam, then I would suggest to include in the system that physicians cannot order such antibiotics for more than 2 days, or for a specific duration, without getting an approval from the responsible ID team”* – Participant 3 *2.7. “A healthcare team should review the patient's records and then decide if the antibiotic should be de-escalated to a narrow spectrum antibiotic”* – Participant 1
Category 4: *Pharmacist’s role*	*2.8. “So with vancomycin, we have to have therapeutic drug monitoring, like measuring trough levels to ensure effective and safe treatment against MRSA”* – Participant 13 *2.9. “I will try to educate the health care teams about the misuse of antibiotics, the disadvantages and the complications. So, they need to learn more about stewardship, its benefits and how they should be more aware of what's better for the patient.”* – Participant 10

### Theme 2: Students’ approach to case interpretation

Students’ clinical reasoning describes the approach that students use to interpret the data, and the strategies they employ through their decision-making processes to arrive at a clinical decision. Participants demonstrated distinct reasoning approaches during case solving. The results reflect the verbalization patterns that students adapted in order to reach a final decision, as demonstrated by the clinical reasoning framework presented in Table 3. These approaches were notably categorized as systematic (quotes 3.1-3.7) vs. non-systematic (quotes 3.8-3.12) approaches to case interpretation and reflected the strategies students used to arrive at decisions.

**Table 3:** Students’ approach to case interpretation

Approach	Generated codes	Reflective quotes
Systematic approach	Critical thinking	*3.1.* “*For empiric therapy, it has to be initiated as soon as possible because it is usually time-sensitive. Empiric therapy would be started within 48 hours according to the* prescriber *on call, but then after 48 hours, any additional dosing required has to be approved by the ID team, which I think is a good approach to optimize empiric therapy first, and then move on to the definitive therapy after the culture results are out.” –* Participant 18
	Three factor approach: Patient-Drug-Bug	*3.2. “First of all, we have to look at the patient, drug and organism factors. The patient has a recurrent urinary tract infection and pyelonephritis, going to the organism, the patient has E.coli but resistant to meropenem, which she is started on empirically, so we can now narrow down to something that covers E.coli.”* – Participant 10 *3.3*. “*The patient had a history of MRSA, and now he is having a central line skin infection that could be caused again by MRSA. Vancomycin covers MRSA. I think Vancomycin is a good option for this patient.”* – Participant 17
	Elimination approach based on sensitivity results and allergy assessment	*3.4. “Third generation cephalosporins is fine for this patient as she cannot take trimethoprim/ sulfamethoxazole because of her sulfa allergy.”* – Participant 5 *3.5.* “*I think I would investigate the sulfa allergy further and if she doesn’t have a true allergy, I would go with trimethoprim/sulfamethoxazole because the organism is sensitive to it and is an oral option. I would step down to oral therapy as soon as she stabilizes.”* – Participant 18
	Evidence/Clinical-based decisions	*3.6.* “*I can look into the Sanford guide or the local Hamad Medical Corporation guidelines to double check my recommendation.”* – Participant 11 *3.7.* “*I would consider general guidelines for my recommendation like the ones from CDC. Also, I will check the local antibiogram.”* – Participant 10
Non-systematic approach	Disturbed thought process	*3.8. “So I will go with giving trimethoprim. Oh no, she is allergic to sulfa. So then I will go with trimethoprim. Oh God why do I keep going back to trimethoprim?”* – Participant 2
	Knowledge deficit/Misinterpretation of questions	*3.9. “I do not know now what the treatment of UTI is. I do not have idea right now. This one I do not recall at all”* – Participant 20
	Lapses and slips (lack of focus)	*3.10. “She is taking meropenem, and the report shows it is sensitive, so mmmm, I think I will keep it”* – Participant 19
	Incertitude	*3.11. “I am not sure how do we actually take effective considerations into account”* – Participant 16 *3.12. “I know that it is a good choice to give Bactrim for the patient, and I know also that cephalosporins can be used. So one of them, I am not sure. OK, maybe I'll go with Bactrim”* – Participant 7

## Discussion

The purpose of this study was to explore students’ cognitive processes when asked to interpret AMS cases. Two themes emerged from data interpretation: were interpreted from students’ focus and students’ clinical reasoning approaches to case interpretation. Students’ focus related to external factors students considered to make and justify therapeutic decisions including patient-centered factors, drug-related factors, AMS interventions, and pharmacist’s role. While students’ clinical reasoning described the approaches to case interpretation and the strategies employed to inform their decision-making processes and arrive at a clinical decision. These included a systematic approach versus non-systematic approach to case interpretation.

Students discussed several important factors and elements that constituted the majority of their focus while solving cases. In fact, those factors constitute the major concept of pharmaceutical care, which aims to optimize drug therapy and ensure patient safety [21]. Also, these findings go along with the ASHP statement on the role of the pharmacist in AMS [22]. Furthermore, participants had consensus that AMS interventions decrease AMR and optimize antimicrobial prescribing [20, 23]. To add, subthemes revealed are considered relevant determinants of ASPs because they go in accordance with the CDC core elements of hospital ASPs [24].

Our findings are consistent with shortcomings of the hypothetic-deductive model, as the latter was developed in the setting of general practice [25]. Yazdani et al recommend a comprehensive model that incorporates specific features of general practice [25]. In our study, the focus is more specific to AMS, so the participants focused more on optimizing antimicrobial regimens and emphasizing on the implementation of AMS interventions. This goes along with outcomes of Gruenberg et al’s study that identified a framinfework for pharmacists’ antimicrobial therapeutic reasoning with emphasis on their role in AMS and in selecting an optimal antimicrobial regimen [26]. For the systematic approach, students were methodical in solving the cases. In fact, the systematic processes adapted by pharmacy students aligned well with previous research that analyzed clinical decision-making of experienced pharmacists in the ambulatory care settings [27]. In addition, students’ clinical reasoning approaches go along with another study that examined physicians’ clinical reasoning while prescribing antimicrobials [18]. Indeed, there were some similarities between the way students and other health care professionals think. However, the main difference was that their results were based on the clinical reasoning of experienced pharmacists, who are exposed to complex patients, in contrast to our study results, which display clinical reasoning of pharmacy students in relation to less complicated patient cases in the scope of AMS [27].

An important cognitive process that few students relied on, but was important nevertheless in guiding their thought process was critical thinking. These particular students were able to analyze the data logically, apply information correctly with justifying their choice and transforming this knowledge according to the context of the case or the patient. Therefore, they were able to reach a clinical recommendation in a systematic way, which suggests the essentiality of critical thinking in effective clinical reasoning and problems solving [28–31]. Additionally, during the think aloud process, while interviewees were trying to navigate their way through the case, some of them supported their answers by clinical guidelines or hospital-specific protocols, while others based it upon their own personal clinical internship experience. Students focused on multiple patient-centered and drug-related factors to approach their case solving. These principal elements constitute basic AMS determinants in pharmaceutical care that are helpful in guiding pharmacists to make a decision [27].

On the other hand, the spontaneous verbalization of thoughts while solving the cases resulted in the adoption of a non-systematic approach during problem-solving. This resulted in the disrupted the student’s train of thought and might have altered their decision-making process. Few students ended up being unorganized, unfocused, unsure, or unaware of what they are actually verbalizing. This has yielded a judgment that either provided an incomplete answer to the given case or did not generate an answer at all. Various cognitive errors can happen throughout the clinical reasoning process that hindered the execution of their verbalization process, including knowledge deficits, slips, or lapses [32, 33]. Specific gaps ID knowledge were identified and could be stressed or re-educated on especially as it pertains to AMS knowledge gap [34]. Incertitude rose as a common non-systematic approach amongst some of the participants in this study, which reflects the hesitancy and indecisiveness in their reasoning processes.

A major strength in our study is using real-time interview to elicit thought process and decision making. However, the research findings should be interpreted in the light of some limitations. We have assumed that a sample size of twenty students is sufficient to generate the expected study outcomes. Despite it being small, we were still able to capture a wide range of cognitive reasoning patterns. This was overcome by assigning three AMS cases to each student rather than one case per student, which will generate more codes and more clinical reasoning processes to interpret. Besides, throughout the process of coding, the co-investigators have identified repetitive or similar codes after analyzing approximately two thirds of the transcripts, which implicates data saturation. This is also supported by other think aloud studies that used quite small sample sizes [35, 36]. Another limitation was that the provided AMS cases were too general for some participants as there were misinterpretations of some questions, which may have disturbed their thought process. However, since our focus is on their thinking process rather than the rightness or wrongness of the answer itself, then this will minimally affect the reliability of our findings. A further limitation was that the study results represent only the cognitive processes of pharmacy students, which does not necessarily represent or reflect the cognitive processes of expert clinical pharmacists in practice. We utilized a prospective think aloud methodology where there is limited possibility for reflection and interpretation of thoughts by the interviewees, thus limiting the errors due to false or incomplete recall, in contrast to other qualitative studies, such as retrospective think aloud and stimulated recall studies which rely on the participant’s recall to reach the research’s objective [37, 38]. Besides, in their study, Ericsson and Simon accentuate that even though some thought processes may be viewed as incomplete, this does not abstain the reliability of the results [39]. In fact, they state that think aloud data obtained from verbal reports are “thoroughly reliable” to reflect the thought process, since they are transcribed verbatim and we believe that robust qualitative results can be derived from inductive thematic data analysis used our study in contrast to deductive thematic analysis [40].

This study has implications for curriculum development and reform. Findings clearly show that students adopt different approaches to decision-making for AMS cases and at times, struggle to apply a systematic reasoning process to the data presented. Educators should focus on developing these skills with students, instead of simply presenting AMS concepts and relying on students to adapt processes developed from other patient care areas. AMS decision-making challenges students by increasing complexity through inclusion of population-level factors (e.g. AMR, institutional antimicrobial susceptibilities) as part of the decision-making process. Students that have these factors as part of their focus, may be able to better implement a systematic approach and arrive at good decisions efficiently. AMS decision-making should therefore be included as part of regular academic programming. Findings helped to explain what students focus on when making decisions, as well as what cognitive strategies they use as part of clinical reasoning during decision-making. These findings add to the understanding of how students approach AMS case data and can be used to direct future educational interventions. Findings also have other implications for practice and research, as described above.

## Conclusions

This study found that students vary in their focus and the cognitive strategies used to interpret AMS cases. Findings support the notion that clinical reasoning and decision-making should be explicitly taught in pharmacy curricula, in order to help students become aware of their own cognitive processes and decision-making abilities. Future research should test the implementation of such programming to determine effective strategies for shifting students to be focused and systematic when approaching AMS case data.

## Data Availability

The datasets used and/or analyzed during the current study are not publicly available due to ethical approval requirement but are available from the corresponding author on reasonable request.

## Abbreviations

AMR: Antimicrobial Resistance
AMS: Antimicrobial Stewardship
ASHP: American Society of Health-System Pharmacists
ASP: Antimicrobial Stewardship Program
CCAPP: Canadian Council for Accreditation of Pharmacy Programs
CDC: Centers for Disease Control and prevention
COVID-19: Coronavirus Disease of 2019
E.coli: Escherichia coli
E.g.: Example
I: Intermediate
ID: Infectious Diseases
IV: Intravenous
MRSA: Methicillin-Resistant Staphylococcus aureus
OK: Okay
PI: Primary Investigator
QU: Qatar University
R: Resistant
S: Sensitive
UTI: Urinary Tract Infection
