# Correction: Investigating pharmacy students’ therapeutic decision-making with respect to antimicrobial stewardship cases

**DOI:** 10.1186/s12909-022-03584-4

**Published:** 2022-06-29

**Authors:** Ziad G. Nasr, Diala Alhaj Moustafa, Sara Dahmani, Kyle J. Wilby

**Affiliations:** 1 College of Pharmacy, QU Health, Qatar University, PO Box 2713, Doha, Qatar; 2 College of Pharmacy, Dalhousie University, Halifax, Canada

**Correction: BMC Medical Education 22, 467 (2022)**

**https://doi.org/10.1186/s12909-022-03542-0**

Following publication of the original article [1], the authors identified an error in the author name of Diala Alhaj Moustafa.

The incorrect author name is: Diala Al Haj Moustafa

The correct author name is: Diala Alhaj Moustafa

The author group has been updated above and the original article [1] has been corrected.
